# Causal effects of circulating inflammatory proteins on COPD: A Mendelian randomization study

**DOI:** 10.1097/MD.0000000000049641

**Published:** 2026-07-03

**Authors:** Huanyu Long, Dian Chen, Lanhe Chu, Simin Jiang, Shurun Li, Congxi Zhang, Yahong Chen

**Affiliations:** a Department of Pulmonary and Critical Care Medicine, Peking University Third Hospital, Beijing, China.

**Keywords:** circulating inflammatory proteins, COPD, Mendelian randomization

## Abstract

Chronic obstructive pulmonary disease (COPD) is a major public health concern due to its high prevalence, morbidity, and mortality. Although COPD is recognized as a systemic inflammatory disease, the specific circulating inflammatory proteins associated with its development and progression remain poorly understood. We performed a Mendelian randomization (MR) study to investigate the association between circulating inflammatory proteins and COPD risk. Genetic data were obtained from a genome-wide association study of 20,066 COPD cases and 338,303 controls from the FinnGen consortium and circulating inflammatory protein data were derived from a genome-wide association study of 14,824 participants. The inverse-variance weighted method was used as the primary analysis. Depending on the number of available instrumental variables, complementary methods including the Wald ratio, Weighted Median, MR-Egger, Weighted Mode, and Simple Mode were applied to assess robustness. Sensitivity analyses were conducted to evaluate heterogeneity and pleiotropy using Cochran’s *Q* test, the MR-Egger intercept, MR-PRESSO, and leave-one-out analysis. In addition, cis-acting protein quantitative trait locus –restricted analyses were performed to further reduce potential pleiotropy. Our findings showed that higher genetically predicted levels of CCL28 (odds ratio [OR] = 0.83, 95% confidence interval [CI]: 0.69–0.99, *P* = .0394), CD40 (OR = 0.94, 95% CI: 0.89–0.99, *P* = .0170), and urokinase-type plasminogen activator (OR = 0.91, 95% CI: 0.85–0.99, *P* = .0212) were associated with a lower risk of COPD, whereas higher levels of Flt3L (OR = 1.09, 95% CI: 1.01–1.18, *P* = .0344) and CD6 (OR = 1.06, 95% CI: 1.02–1.12, *P* = .0099) were associated with a higher risk. Sensitivity analyses showed no evidence of heterogeneity or directional pleiotropy, and leave-one-out analyses indicated that the results were not driven by any single nucleotide polymorphism. These findings suggest that circulating inflammatory proteins, including CCL28, CD40, urokinase-type plasminogen activator, Flt3L, and CD6, may be involved in COPD pathogenesis. Further studies are needed to validate these findings and clarify their potential biological relevance.

## 1. Introduction

Chronic obstructive pulmonary disease (COPD) is a heterogeneous lung condition characterized by chronic inflammation and persistent airway obstruction, leading to progressive and irreversible decline in lung function.^[1,2]^ Globally, COPD affects approximately 294 million people and is responsible for more than 3 million deaths each year.^[3,4]^ It currently ranks as the fourth leading cause of death worldwide and is projected to rise even further in the coming decades.^[5]^ With an aging global population, both the incidence and mortality of COPD continue to rise, contributing to a major public health challenge and imposing a substantial socioeconomic burden.^[6]^

Systemic inflammation is known to be associated with COPD, but the underlying mechanisms remain poorly understood.^[7]^ Cigarette smoke, as a major risk factor, can induce inflammatory responses in the lungs that may extend into the circulation and contribute to systemic inflammation.^[8]^ Observational studies have reported elevated levels of circulating inflammatory proteins in patients with COPD, including interleukins such as IL-1β and IL-6, tumor necrosis factor (TNF)-alpha and C-reactive protein.^[9]^ These proteins have been linked to disease severity, more frequent exacerbations, and accelerated lung function decline.^[9]^ However, such associations do not confirm causality and may be influenced by confounding factors.

To address these limitations, we applied Mendelian randomization (MR), which uses genetic variants such as single nucleotide polymorphisms (SNPs) identified from genome-wide association studies (GWAS) as instrumental variables (IVs) to infer causal relationships between exposures and outcomes.^[10,11]^ Since genetic variants are randomly allocated at conception and generally not affected by environmental or lifestyle factors, MR can minimize confounding and reverse causality inherent in traditional observational studies.^[12]^ In this study, we employed a 2-sample MR approach to assess the causal effects of circulating inflammatory proteins on COPD risk. This approach aimed to clarify their potential role in COPD pathogenesis and to identify candidate biomarkers or therapeutic targets for early intervention.

## 2. Methods

### 2.1. Study design

**Figure 1.**
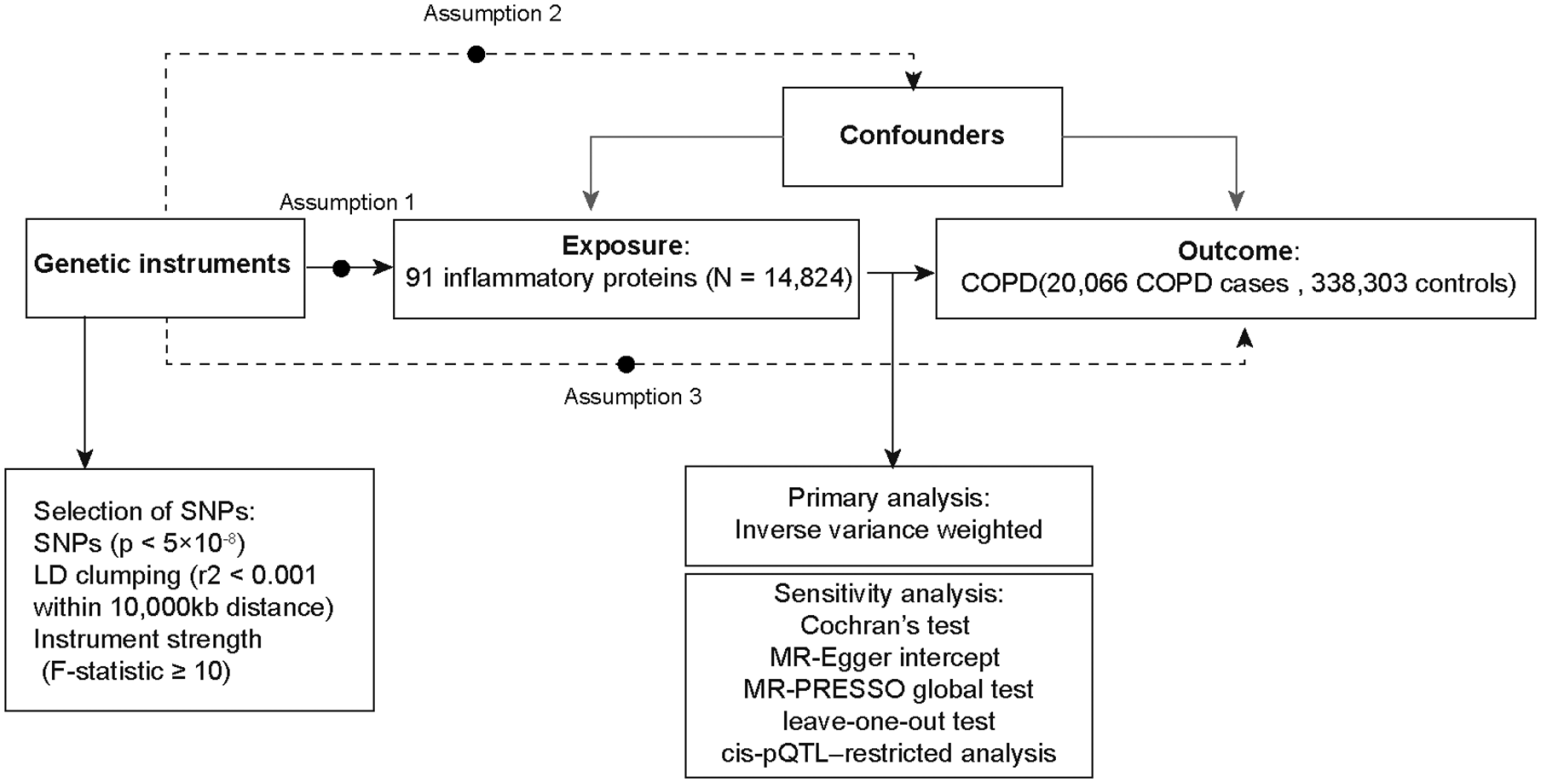
Overview of this Mendelian randomization analysis. Assumption 1: genetic instruments are strongly associated with the exposures of interest. Assumption 2: genetic instruments are independent of confounding factors. Assumption 3: genetic instruments are not associated with the outcome and affect the outcome only via exposures. IVW = inverse-variance weighted; LD = linkage disequilibrium; SNPs = single nucleotide polymorphisms; COPD = chronic obstructive pulmonary disease.

The overall study design is presented in Figure 1. This 2-sample MR study was conducted in accordance with the STrengthening the Reporting of OBservational studies in Epidemiology using Mendelian randomisation reporting guidelines.^[13]^ Summary-level genetic association data for 91 circulating inflammatory proteins were obtained from a meta-analysis of 11 independent cohorts, comprising a total of 14,824 participants.^[10]^ Plasma protein levels were measured using the Olink Target-96 inflammation panel, and genome-wide protein quantitative trait loci mapping was performed. The resulting summary statistics for these proteins are publicly available through the GWAS Catalog (https://www.ebi.ac.uk/gwas/) under accession numbers GCST90274758 to GCST90274848. For the outcome data, we used summary-level statistics for COPD from the FinnGen consortium (https://www.finngen.fi/en), specifically from the most recent data release (R10).^[14]^ This dataset includes 20,066 COPD cases (coded as R10_J10_COPD based on international classification of diseases criteria) and 338,303 controls, with diagnoses derived from nationwide Finnish health registries. As this study exclusively used publicly available, de-identified summary data, no additional ethical approval or informed consent was required. Ethical approval was not required for this study because all data were obtained from publicly available GWAS with no access to individual-level data. All original studies included in the GWAS datasets had received ethical approval from their respective institutional review boards, and all participants had provided written informed consent.

### 2.2. Selection of IVs

To identify IVs, we applied the following criteria: SNPs were required to exhibit genome-wide significant associations with each circulating inflammatory protein (*P* < 5 × 10^−8^); SNPs had to be independently associated with the exposure, with linkage disequilibrium defined as *r*^2^ <0.001 and a distance >10,000 kb to ensure independence among instruments. To ensure sufficient instrument strength for causal inference, we evaluated each SNP using the *F* statistic, calculated as *F* = β^2^/ SE^2^, where β represents the effect size and SE is the standard error. SNPs with *F* < 10 were excluded due to the risk of weak instrument bias.^[15]^ A higher *F* statistic reflects stronger instrument strength and a lower likelihood of introducing bias into the MR estimates. To confirm the direction of causality, we also performed the Steiger directionality test, which compares the proportion of variance explained in the exposure and the outcome to validate the assumed causal direction.

### 2.3. MR analysis

The validity of MR analysis relies on 3 core assumptions: relevance, where genetic variants are strongly associated with the exposure; independence, where genetic variants are independent of confounders; and exclusion restriction, where genetic variants influence the outcome only through the exposure. Additional assumptions for sensitivity analyses include the absence of directional pleiotropy. In this study, the inverse-variance weighted (IVW) method was employed as the primary approach to estimate the causal relationship between circulating inflammatory proteins and COPD.^[16]^ To complement the main analysis, Weighted Median, MR-Egger, Weighted Mode, and Simple Mode were also applied.^[17–19]^ The selection of MR methods was based on the number of available IVs: the Wald ratio was used when only 1 SNP was available, the IVW method was applied when 2 SNPs were available, and additional methods such as MR-Egger and mode-based approaches were used when 3 or more SNPs were available. Causal estimates were reported as odds ratios with 95% confidence intervals. Nominal statistical significance was defined as *P* < .05.^[20]^ When there was no evidence of heterogeneity or horizontal pleiotropy, the IVW method was considered the most appropriate.

### 2.4. Sensitivity analysis

To evaluate the robustness of the MR findings, several sensitivity analyses were performed. Heterogeneity among SNPs was assessed using Cochran’s *Q* statistic under the IVW model, with *P* > .05 indicating no evidence of significant heterogeneity. To test for horizontal pleiotropy, the MR-Egger intercept was calculated, where a nonsignificant intercept (*P* > .05) suggests limited evidence of directional pleiotropy. We further applied the MR-PRESSO global test to detect potential outlier variants that may bias the estimates. This test was conducted when the number of IVs was greater than or equal to 4, in accordance with its methodological assumptions. For exposures with fewer than 4 IVs, MR-PRESSO was not applicable, and results were interpreted with caution. A leave-one-out analysis was conducted by sequentially excluding each SNP to evaluate whether the causal association was disproportionately influenced by any single SNP. In addition, to further address potential pleiotropy, we performed a sensitivity analysis restricted to cis-acting protein quantitative trait locus (cis-pQTLs) (variants located within ±1 Mb of the encoding gene), which are less likely to exert pleiotropic effects. All statistical analyses were performed using R software (version 4.5.1) in RStudio (version 2025.09.2+418; Posit Software, PBC). MR analyses were conducted using the TwoSampleMR package (version 0.6.15; MRC Integrative Epidemiology Unit, University of Bristol) and MR-PRESSO package (version 1.0; Marie Verbanck et al).

## 3. Results

### 3.1. Selection of IVs

Detailed information on the 91 circulating inflammatory proteins is provided in Table S1, Supplemental Digital Content 1. Among these, 5 inflammatory proteins were selected for further MR analysis based on their potential associations with COPD risk. After quality control, a total of 26 SNPs associated with the 5 selected inflammatory proteins were identified and used as IVs in the MR analysis. Specifically, C-C motif chemokine 28 (CCL28) was linked to 4 SNPs, CD40L receptor (CD40) to 3 SNPs, T-cell surface glycoprotein CD6 isoform (CD6) to 3 SNPs, fms-related tyrosine kinase 3 ligand (FIt3L) to 9 SNPs, and urokinase-type plasminogen activator (uPA) to 7 SNPs. The detailed SNP information used as IVs is presented in Table S2, Supplemental Digital Content 2. All selected IVs had *F* > 10, indicating strong instrument strength and minimizing the risk of weak instrument bias. Additionally, all instruments passed the Steiger directionality test, supporting a unidirectional causal effect of inflammatory protein levels on COPD risk (Table S2, Supplemental Digital Content 2).

### 3.2. Causal effects of circulating inflammatory proteins on COPD

**Figure 2.**
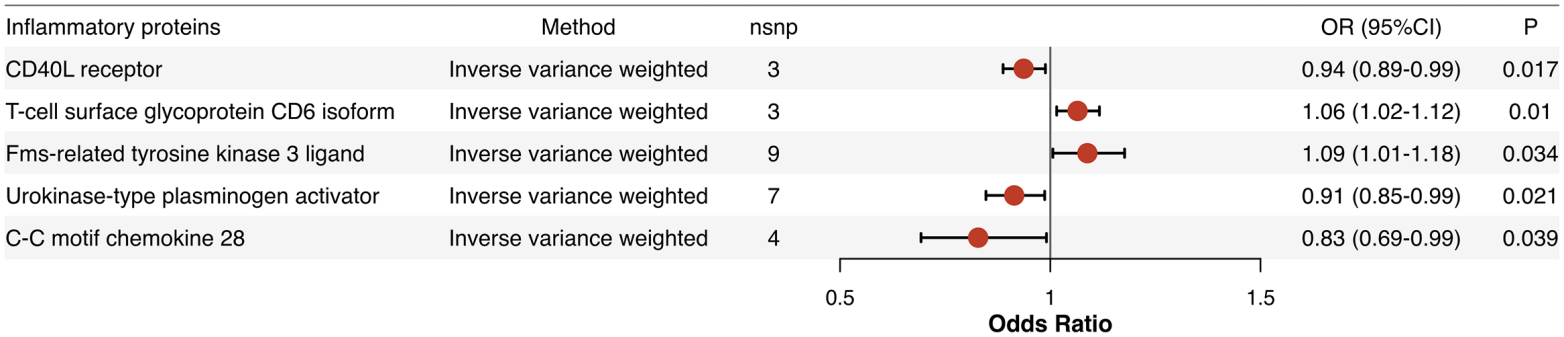
Forest plots showing causal relationships between circulating inflammatory proteins and COPD. OR = odds ratio; CI = confidence interval; COPD = chronic obstructive pulmonary disease; SNP = single nucleotide polymorphism.

Based on the IVW method, genetically predicted higher levels of CCL28 (odds ratio [OR] = 0.83, 95% confidence interval [CI]: 0.69–0.99, *P* = .0394), CD40 (OR = 0.94, 95% CI: 0.89–0.99, *P* = .0170), and uPA (OR = 0.91, 95% CI: 0.85–0.99, *P* = .0212) were associated with a reduced risk of COPD. In contrast, elevated genetically predicted levels of FIt3L (OR = 1.09, 95% CI: 1.01–1.18, *P* = .0344) and CD6 (OR = 1.06, 95% CI: 1.02–1.12, *P* = .0099) were associated with an increased risk of COPD (Fig. 2). Additional results obtained using the other methods are provided in Table S3, Supplemental Digital Content 3.

### 3.3. Sensitive analysis

**Figure 3.**
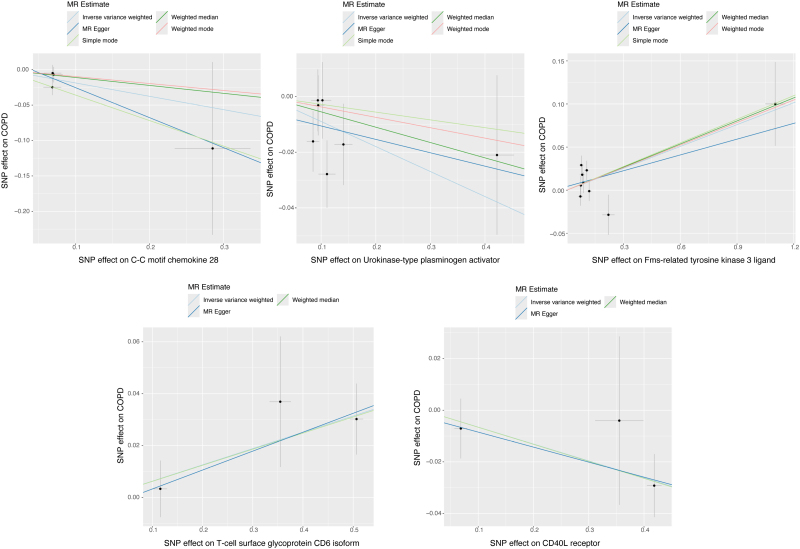
Scatter plot for the relationship between circulating inflammatory proteins and COPD. COPD = chronic obstructive pulmonary disease; SNP = single nucleotide polymorphism; MR = Mendelian randomization.

**Figure 4.**
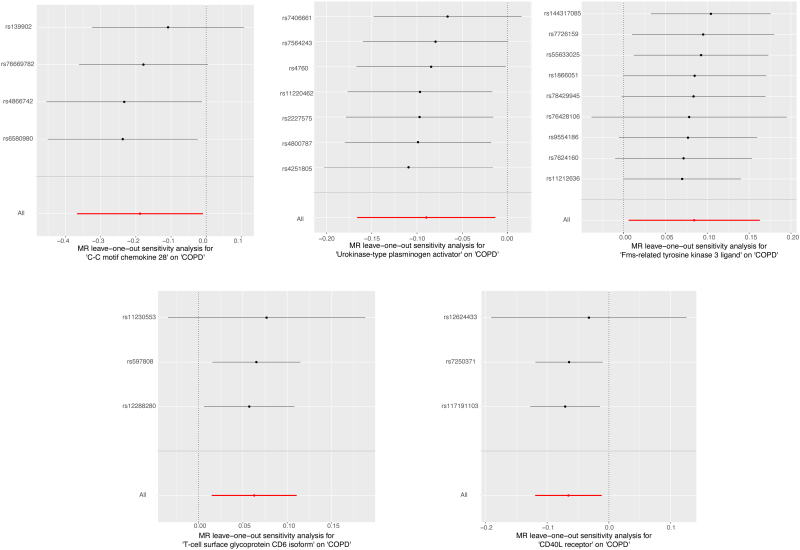
Funnel plot for the relationship between circulating inflammatory proteins and COPD. COPD = chronic obstructive pulmonary disease; MR = Mendelian randomization.

Sensitivity analyses were performed to assess the robustness of the MR findings. Cochran’s *Q* test based on the IVW model showed no evidence of significant heterogeneity among the SNPs (Table S4, Supplemental Digital Content 4). Consistently, the MR-Egger intercept test suggested limited evidence of directional horizontal pleiotropy. The MR-PRESSO global test was conducted for exposures with 4 or more instrumental variables and did not detect evidence of outlier variants. Visual inspection of scatter plots did not indicate obvious outliers or substantial deviations from the overall trend (Fig. 3). Leave-one-out analyses showed that no single SNP disproportionately influenced the overall causal estimates, supporting the stability of the results (Fig. 4). In addition, sensitivity analyses restricted to cis-pQTLs were performed to further address potential pleiotropy. In the cis-restricted analysis, some associations (e.g., CD6 and CD40) remained consistent in both direction and statistical significance, whereas others showed attenuation or loss of statistical significance (e.g., CCL28 and uPA), although the overall direction of effect was generally preserved (Table S5, Supplemental Digital Content 5).

## 4. Discussion

COPD has been linked to various factors, but how these factors are associated with the disease remains unclear. Whether circulating inflammatory proteins play a causal role in COPD pathogenesis or simply reflect the underlying inflammatory state is still unclear. To explore this, we conducted an MR analysis, which uses genetic variants as IVs to assess causal relationships while minimizing confounding and reverse causality. In this study, we systematically examined 91 inflammatory proteins, including growth factors, interleukins, TNFs, signaling molecules, and chemokines, as potential causal exposures for COPD. In the primary analysis, several proteins showed nominal associations with COPD risk. Specifically, higher genetically predicted levels of CD40, CCL28 and uPA were associated with a reduced risk of COPD, whereas elevated levels of CD6 and FIt3L were associated with an increased risk. Overall, these findings suggest that circulating inflammatory proteins may be involved in COPD pathogenesis, while also highlighting the complexity of immune regulation in the disease. Further studies are required to validate these associations and clarify their biological relevance.

First, CD6 and CD40 showed relatively consistent evidence across analyses, including concordant effect directions and support from cis-pQTL–restricted sensitivity analyses, suggesting a more robust potential involvement in COPD pathogenesis. CD6 and CD40 are both involved in T-cell signaling and immune regulation, yet they appear to exert opposing effects on COPD risk in our MR analysis. CD6 is a type I transmembrane glycoprotein primarily expressed on T cells, where it interacts with ligands such as ALCAM (CD166) and CD318.^[21]^ As a member of the scavenger receptor cysteine-rich superfamily, CD6 plays a regulatory role in lymphocyte activation and differentiation.^[22]^ Increased CD6 signaling has been associated with enhanced T-cell activation and chronic inflammatory responses, particularly in autoimmune diseases such as rheumatoid arthritis and multiple sclerosis.^[23]^ In the context of COPD, sustained T-cell activation may contribute to persistent airway inflammation and tissue damage, which is consistent with our finding that genetically predicted higher CD6 levels were associated with increased COPD risk. In contrast, CD40, a member of the TNF receptor superfamily, is expressed on a wide range of immune and structural cells, including endothelial cells, fibroblasts, and epithelial cells. Through interaction with CD40L on activated CD4^+^ T cells, CD40 signaling is involved in both immune activation and tissue regulation.^[24]^ Although CD40 is generally considered pro-inflammatory in acute immune responses, emerging evidence suggests that it may exert context-dependent, potentially protective effects in chronic disease settings. For example, CD40 activation in dendritic cells has been reported to attenuate atherosclerosis by reducing lipid uptake.^[25]^ In the lung, CD40 expression has been implicated in remodeling processes, and reduced CD40 levels have been observed in COPD-associated pulmonary hypertension.^[26]^ These findings support a potential protective role of CD40 in chronic pulmonary inflammation, in line with our MR results showing that higher genetically predicted CD40 levels were associated with a lower risk of COPD.

Chemokines are chemoattractant cytokines that play a central role in coordinating innate and adaptive immune responses.^[27]^ Structurally, chemokines are characterized by the presence of 4 conserved cysteine residues that determine their 3-dimensional configuration.^[28]^ Based on the relative positions of these cysteine residues, chemokines are classified into 4 major subfamilies: CXC (α-chemokines), CC (β-chemokines), C (γ-chemokines), and CX3C (δ-chemokines).^[29,30]^ In our study, we identified 1 chemokine, CCL28 (a CC chemokine), whose genetically predicted circulating levels showed a nominal association with COPD risk. CCL28, also known as mucosae-associated epithelial chemokine, is constitutively expressed in mucosal epithelial tissues such as the respiratory tract. It primarily signals through CCR10 and CCR3, mediating the homing of CCR10^+^ T and B cells and promoting eosinophil migration.^[31]^ In addition to its chemotactic functions, CCL28 has been reported to exhibit broad-spectrum antimicrobial activity.^[29]^ This dual role in immune cell recruitment and microbial defense suggests that CCL28 may contribute to maintaining mucosal barrier integrity and limiting pathogen-induced inflammation in the airway. In our MR analysis, higher genetically predicted circulating levels of CCL28 were associated with a lower risk of COPD, suggesting a potential protective role in preserving epithelial function and modulating airway immune responses. In our MR analysis, higher genetically predicted circulating levels of CCL28 were associated with a lower risk of COPD. However, this association was attenuated in the cis-restricted analysis and should therefore be interpreted with caution, despite a consistent direction of effect.

uPA is a serine protease involved in tissue remodeling and immune regulation.^[32]^ In our MR analysis, higher genetically predicted levels of uPA were associated with a lower risk of COPD. However, this association was attenuated in the cis-restricted analysis and should therefore be interpreted with caution, despite a consistent direction of effect. Dysregulated uPA signaling – particularly through the vitronectin-uPA axis – has been implicated in airway and parenchymal remodeling in COPD. Experimental studies suggest that disruption of this pathway may exacerbate emphysema and inflammation, whereas restoration of uPA function may help preserve lung structure.^[33]^ Although its receptor product soluble urokinase plasminogen activator receptor is often elevated in COPD and reflects inflammatory activity, uPA itself may exert context-dependent reparative effects.^[34]^ These findings suggest that a balanced uPA/uPAR axis may be important for pulmonary homeostasis. In addition, Flt3L showed a nominal association in the primary analysis, but the lack of supporting cis-pQTL instruments limits further causal interpretation.

This study has several limitations. Although MR reduces confounding and reverse causality, residual pleiotropy cannot be fully excluded, particularly given the inclusion of trans-acting protein quantitative trait locus, despite additional sensitivity analyses including cis-restricted analyses. Moreover, none of the associations remained statistically significant after multiple testing correction and should therefore be interpreted as exploratory. In addition, the limited number of instrumental variables for some proteins restricted the application of certain MR methods. Finally, the use of summary-level data and predominantly European populations may limit subgroup analyses and generalizability. Further validation in independent and diverse cohorts is warranted.

## 5. Conclusion

In conclusion, this MR study provides insights into the potential roles of circulating inflammatory proteins in COPD. Proteins including CCL28, CD40, uPA, Flt3L, and CD6 may be involved in disease pathogenesis, although these associations should be interpreted with caution. Overall, our findings highlight the complexity of immune regulation in COPD and warrant further experimental and clinical validation.

## Author contributions

**Conceptualization** – Huanyu Long, Yahong Chen.

**Data curation** – Huanyu Long, Dian Chen, Yahong Chen.

**Methodology** – Huanyu Long, Dian Chen, Simin Jiang, Shurun Li.

**Writing** – **original draft:** Huanyu Long.

**Writing** – **review & editing:** Dian Chen, Lanhe Chu, Simin Jiang, Shurun Li, Congxi Zhang, Yahong Chen.

**Formal analysis** – Lanhe Chu.

**Software** – Congxi Zhang.

**Funding acquisition** – Yahong Chen.

**Supervision** – Yahong Chen.










